# The state of diabetes care and obstacles to better care in Aceh, Indonesia: a mixed-methods study

**DOI:** 10.1186/s12913-023-09288-9

**Published:** 2023-03-20

**Authors:** Hizir Sofyan, Farah Diba, Suryane S. Susanti, Marthoenis Marthoenis, Ichsan Ichsan, Novi Reandy Sasmita, Till Seuring, Sebastian Vollmer

**Affiliations:** 1 Universitas Syiah Kuala, Banda Aceh, Indonesia; 2 Universitas Indonesia, Depok, Indonesia; 3 Luxembourg Institute of Socio-Economic Research (LISER), Esch-Sur-Alzette, Luxembourg; 4 University of Goettingen, Göttingen, Germany

**Keywords:** Diabetes, Health systems, Indonesia

## Abstract

**Background:**

Cardio-metabolic diseases are a major cause of death worldwide, including in Indonesia, where diabetes is one of the most critical diseases for the health system to manage.

**Methods:**

We describe the characteristics, levels of control, health behavior, and diabetes-related complications of diabetes patients in Aceh, Indonesia. We use baseline data and blood testing from a randomized-controlled trial. We conducted semi-structured interviews with eight health providers from Posbindu and Prolanis programs that target diabetes and other non-communicable diseases (NCDs). We also conducted three focus group discussions with 24 diabetes patients about their experiences of living with diabetes and the existing support programs.

**Results:**

The blood tests revealed average HbA1c levels indicative of poor glycemic control in 75.8 percent of patients and only 20.3 percent were free from any symptoms. Our qualitative findings suggest that patients are diagnosed after diabetes-related symptoms manifest, and that they find it hard to comply with treatment recommendations and lifestyle advice. The existing programs related to NCDs are not tailored to their needs.

**Conclusion:**

We identify the need to improve diabetes screening to enable earlier treatment and achieve better control of the disease. Among diagnosed patients, there are widespread beliefs about diabetes medication and alternative forms of treatment that need to be addressed in a respectful dialogue between healthcare professionals and patients. Current diabetes screening, treatment and management programs should be revised to meet the needs of the affected population and to better respond to the increasing burden of this disease.

## Introduction

In Indonesia, diabetes prevalence is 8.2%, slightly above the average of 7.5% for all low- and middle-income countries (LMICs) [1]. Although these rates are similar to those in Western societies, many LMIC health systems are not well prepared to deal with this increased burden of diabetes. Manne-Goehler et al. (2019) found, for a sample of 28 LMICs, that less than half of all people living with diabetes have been diagnosed [2]. While most diagnosed people receive some sort of treatment, only half are adequately controlled. In Indonesia, about 21% of people living with diabetes are diagnosed and just one-third of them (7% of all people living with diabetes) are adequately controlled.

This health burden also has economic implications. Bommer et al. (2017) estimate that the global cost of diabetes in 2015 was 1.3 trillion US dollars, which is equivalent to 1.8% of global GDP [3]. In Indonesia, direct costs were around 3.6 billion US dollars, equivalent to 0.4% of Indonesia’s GDP and 13.7% of Indonesia’s total health expenditure. The losses to the labor market were of similar magnitude, bringing the total cost of diabetes to 0.8% of GDP. This highlights the importance of reducing the number of new diabetes cases and improving care for people living with diabetes, from both a quality of life and an economic perspective. This study examines how to improve the treatment of people who already have a diabetes diagnosis.

Indonesia has several public programs to reduce the burden of NCDs, including health screenings, integrated management of diabetes and hypertension at public health posts (Puskesmas), screening for breast and cervical cancer, smoking cessation programs, increasing physical activity, and healthy eating [4]. Two widely-implemented programs at the primary care level, *Posbindu* and *Prolanis*, focus on the early detection of cardio-metabolic diseases and the prevention of secondary complications. Posbindu is a program that runs at Puskesmas and focuses on early detection, prevention and monitoring of NCD risk factors within their communities. It targets at-risk groups and people with NCDs aged 15 years and more. Prolanis is a proactive health care program implemented in an integrated way involving participants, Puskesmas, and the public health insurance system (BPJS). It aims to achieve an optimal quality of life with cost-effective and efficient health services for patients with chronic diseases. While Posbindu focuses on NCDs of any kind, *Prolanis* focuses on people with diabetes or hypertension. While these programs are intended to be universally accessible under universal health coverage, little is known about their efficacy [5] (see Table 1 for more information about Prolanis and Posbindu).

**Table 1:** Description of existing chronic disease programs

**Posbindu**
Every Puskesmas in Indonesia hosts the Posbindu PTM program, which is implemented by the Indonesian Ministry of Health. Each Puskesmas has a dedicated Posbindu PTM program manager who is responsible for the treatment of patients with a NCD, including type 2 diabetes. Because Puskesmas cover relatively large geographical areas, especially in rural regions, the Posbindu program is provided at village level to increase the accessibility of health services. Usually, one doctor, two nurses, and local community health workers from the villages attend monthly meetings and provide health examinations, counselling, and health education, as well as basic physical examinations
**Prolanis**
Similar to Posbindu PTM, all Puskesmas in Banda Aceh and Aceh Besar offer the Prolanis program. Prolanis was designed by the Indonesian National Health Insurance Organization (BPJS Kesehatan) specifically for patients with diabetes and hypertension. At each Puskesmas, a group of at least 30 patients with diabetes or hypertension is established. Prolanis offers weekly activities at the Puskesmas and this includes physical exercise. Unlike Posbindu, all Prolanis activities are held centrally at Puskesmas. Each Prolanis group is supported by a team consisting of one nurse (who could be a Posbindu PTM program manager) who functions as the Prolanis program manager, one doctor and one additional nurse. Each Puskesmas is free to choose the staff involved in the program

We use a mixed-methods approach to better understand the characteristics and experiences of people living with diabetes in Aceh province in Indonesia. We do a descriptive investigation of the characteristics of patients with type 2 diabetes, based on data recently collected in Banda Aceh and Aceh Besar. We then investigate how the Posbindu and Prolanis programs function and issues related to their implementation, using information from qualitative interviews with program managers and participants.

## Methods

To identify the current health status of people with diabetes in Aceh province, we explore baseline data from a randomized controlled trial (RCT) conducted in early 2019. The sample was drawn from people who had a diagnosis of Type 2 diabetes at primary health posts in Banda Aceh and Aceh Besar [6]. This RCT investigated the effect of peer education sessions for type 2 diabetes on diabetes-related outcomes in the province of Aceh within local primary health care posts. Criteria for inclusion in the RCT were (1) treated for type 2 diabetes in the Puskesmas of Banda Aceh or Aceh Besar and (2) aged between 20 and 89 years. Puskesmas staff contacted people on their patient lists to recruit them to this study. A total of 533 participants were recruited and baseline data were collected in March and April 2019. We obtained written consent from the patients. Patients were informed that they could drop out of the study at any point. Further details about the data collection can be found in Seuring et al. (2019) [6].

521 participants had non-missing data on age, education, and relevant biomarkers. The statistical analysis is limited to the calculation of averages, standard deviations, and frequencies. Poor glycemic control is defined as a HbA1c level greater than or equal to 64 mmol/l; high cholesterol is defined as a total cholesterol level greater than or equal to 6.2 mmol, and hypertension is defined as systolic blood pressure above 130 and diastolic blood pressure above 80. In addition to providing estimates of lab-based measures of HbA1c, cholesterol and BMI (based on measured waist and height) to assess the metabolic profile of type 2 diabetes patients in Aceh and self-reported information on prevalent diabetes complications, we also use information on diabetes-related distress, based on the two-item diabetes distress scale (DDS2) [7]. The DDS2 has been validated and is considered a good alternative to longer, more extensive scales such as the DDS17 [7]. The DDS2 measures diabetes-distress based on two questions with a 6-item Likert response scale, going from 1 (no problem) to 6 (serious problem). The questions are:

1. Feeling overwhelmed by the demands of living with diabetes.
2. Feeling that I am often failing with my diabetes regimen.

An average score of ≥ 3 was considered to be indicative of diabetes-specific distress, following the recommendations of Fisher et al. (2008) [7]. For physical activity we calculated a binary measure of whether participants achieved World Health Organisation (WHO)-recommended levels of physical activity. The threshold is > 600 min per week of total physical activity metabolic equivalent of task (MET), using data collected with the Global Physical Activity Questionnaire (GPAQ).

We conducted semi structured individual interviews with eight health officers from the Aceh Provincial Health Office. The interviews were conducted in eight Puskemas with a high share of type 2 diabetes patients. Two Puskesmas were located in Banda Aceh and six in Aceh Besar. The interviews were designed to ascertain the level of engagement with the current NCD programs and activities conducted in Puskesmas.

Following the interviews, diabetes patients from these Puskesmas were invited to participate in focus group discussions (FGDs). Three FGDs were conducted with 24 patients in two Puskesmas in Aceh Besar and one Puskesmas in Banda Aceh. The FGDs sought information on patients’ understanding of diabetes and their motivation for, and feelings about, participating in the Posbindu PTM and Prolanis programs, and explored their experiences of receiving diabetes treatment in Puskesmas.

The sampling strategy for interviews and FGDs was purposive sampling. The inclusion criteria for interviews were: health officer of Puskesmas in Banda Aceh or Aceh Besar, who is responsible for Prolanis and Posbindu, and had at least one year of work experience. The inclusion criteria for FGDs were: patients diagnosed with diabetes from Banda Aceh or Aceh Besar, who actively participated in the Posbindu and Prolanis activities, and who were at least 18 years old. We obtained the contact details of diabetes patients from Prolanis and Posbindu staff. The interviews and FGDs were conducted face-to-face by study authors FD and SSS, and lasted approximately one hour. With participants’ permission, the interviews and FGDs were recorded on an encrypted laptop or audio recording device and then transcribed verbatim by the research team. The interviews and the FGDs were conducted in Bahasa Indonesia and later translated to English after transcripts were anonymized and all names were replaced by identifier numbers. We then conducted line by line analysis and utilized inductive thematic analysis to code the transcripts and cluster them into themes, along with anonymized quotes to support the themes, for presentation in the final report.

## Results

### Descriptive Statistics

Our descriptive results are stratified by men and women, with men being older and having higher formal education levels (Table 2). The results show very high average HbA1c levels of 84 mmol/l (9.8%), well above common thresholds for uncontrolled diabetes of either 53 mmol/l (7%), which is also the target HbA1c level recommended by the Indonesian Society of Endocrinology (PERKENI) T2DM guidelines, or 75 mmol/l (9%) [8]. The majority of women and around 45% of men were either overweight or obese. About one-quarter of the population had blood pressure levels indicative of hypertension and 25% of men and 34% of women had high total cholesterol levels.

**Table 2:** Characteristics of patients diagnosed with diabetes in Aceh, Indonesia

	Male	Female	
	(*N* = 95)	(*N* = 426)	*p*-value
**Age**			
Mean (SD)	58.12 (8.92)	52.83 (10.17)	< 0.001
**Highest level of education**			
Less than primary	1 (1.1%)	28 (6.6%)	< 0.001
Primary	21 (22.1%)	135 (31.7%)	
Secondary	51 (53.7%)	223 (52.3%)	
Post-secondary	22 (23.2%)	40 (9.4%)	
**HbA1c (mmol/l)**			
Mean (SD)	85.18 (28.75)	84.47 (26.03)	0.814
**Poor glycemic control (HbA1c > = 64 mmol/l)**			
No	25 (26.3%)	99 (23.2%)	0.524
Yes	70 (73.7%)	327 (76.8%)	
**Total cholesterol (mmol/l)**			
Mean (SD)	5.42 (1.45)	5.67 (1.26)	0.087
**BMI**			
Mean (SD)	24.99 (4.55)	25.73 (4.59)	0.151
**Waist circumference in cm**			
Mean (SD)	93.89 (9.99)	93.41 (10.60)	0.686
**Hypertension (syst. > 130 & diast. > 80)**			
Normal blood pressure	69 (72.6%)	330 (77.5%)	0.314
High blood pressure	26 (27.4%)	96 (22.5%)	
**Cholesterol > = 6.2 mmol**			
Normal cholesterol	71 (74.7%)	283 (66.4%)	0.117
High cholesterol	24 (25.3%)	143 (33.6%)	
**BMI categories**			
Normal weight	52 (54.7%)	200 (46.9%)	0.337
Overweight	33 (34.7%)	164 (38.5%)	
Obese	10 (10.5%)	62 (14.6%)	
**NCD program participation**			
No	41 (43.2%)	94 (22.1%)	< 0.001
Prolanis	19 (20.0%)	90 (21.1%)	
Posbindu	35 (36.8%)	242 (56.8%)	
**High diabetes distress**			
No to moderate distress	26 (27.4%)	115 (27.0%)	0.941
High distress	69 (72.6%)	311 (73.0%)	
**Meets WHO recommended:MIC physical activity levels**			
No	36 (58.1%)	189 (65.9%)	0.245
Yes	26 (41.9%)	98 (34.1%)	
**Has diabetes complications**			
No complications	14 (16.1%)	82 (21.5%)	0.367
Rethinopathy	26 (29.9%)	121 (31.7%)	
Kidney disease	3 (3.4%)	6 (1.6%)	
Impaired wound healing	7 (8.0%)	22 (5.8%)	
Neurological problems	16 (18.4%)	77 (20.2%)	
Cardio-vascular disease	18 (20.7%)	51 (13.4%)	
Diabetic foot	3 (3.4%)	23 (6.0%)	

Number of observations for WHO physical activity levels was 349, for diabetes complications 469. All other variables had no missing observations

Only 20% of men and women in our sample reported being free from diabetes-related complications. The most prevalent complication directly attributable to diabetes was retinopathy, with every third respondent reporting diabetes-related eye complications. Severe complications, such as neurological problems that impair pain sensitivity and slow wound healing, were reported more frequently, but there were fewer reports of very severe diabetes complications, such as diabetic foot and kidney disease.

Measures of lifestyle and preventive behavior in this population showed that while men were still quite likely to achieve high levels of physical activity throughout the day, these rates were much lower for women. Women, however, were more likely to participate in NCD prevention and treatment programs.

Results from the two-item diabetes distress scale showed that the majority of participants reported high levels of diabetes distress resulting from being overwhelmed by the disease and its management, and a feeling of failure in its management [7]. High diabetes distress has been related to adverse effects, including less successful disease management and depression [7].

### Qualitative results

This section provides the results of the qualitative interviews with Posbindu and Prolanis program managers and FGDs with diabetes patients. Qualitative data were analyzed based on the results of interviews with eight health officers and FGDs with 24 diabetes patients (16 women and 8 men) with a mean age of 52 (range of 28–74 years). The results of the data analysis are grouped into themes and sub-themes that emerged during the analysis. The two main themes were: (1) the difficulty of reaching and engaging patients and (2) experiences of diabetes patients. We grouped the sub-themes, consisting of challenges faced by health officers and patients, within these main themes.

#### Theme 1: The difficulty of reaching and engaging patients

The health officers responsible for Posbindu and Prolanis often mentioned that it was challenging to motivate patients to participate in the programs beyond basic laboratory tests (e.g. blood sugar, uric acid, and cholesterol blood tests).

“*I don’t know what to say…. most of the time, there is little interest in attending the Posbindu sessions because the participants find them boring. … They are less motivated to attend sessions with physical activity and health education… unless we come to the village (Posbindu post) with the drugs or blood sugar, cholesterol or uric acid tests… sometimes they do not want to attend if we only offer blood sugar tests… because they want to have another test (cholesterol, uric acid).*” (PAB1)

Another challenge was to reach the working-age population, as activities took place during general working hours.

“*The problem is that we conduct the Posbindu activities during working hours, and therefore cannot reach those of working age but only elderly people… because those of working age are usually at work or attend school during the Posbindu activity in the village… we therefore cannot attend to many patients of younger age, despite our records showing that we have diabetes patients in their thirties….*” (PBA1)

The Prolanis program required a minimum of 30 attendees per session. It was often difficult to reach this number, because people were unwilling to come to Puskesmas each time or they had other commitments. Puskesmas would then bring in other patients to meet BPJS Kesehatan’s minimum number of 30 diabetes and hypertension patients. This meant that, although the number of attendees was stable, the composition of the group was different for each session.

“*We have 30 diabetes and hypertension patients in our Prolanis club, however, the participants are constantly changing… because not all 30 participants attend the sessions every month… hence we keep changing the participants since the BPJS required at least 30 patients in each session.*” (PAB4)

#### Theme 2: Experiences of diabetes patients

Three focus group discussions (FGD) were conducted with diabetes patients in Banda Aceh and Aceh Besar to understand their experiences of living with diabetes. The respondents were predominantly women who reported homemaking as their occupation, and were mostly between 50 and 55 years of age. Many reported having experienced symptoms such as impaired wound healing or fatigue, frequent urination, and thirst that forced them to visit the hospital, where they were diagnosed with diabetes.

*“I found out that I had DM when I had a wound that never healed. It was in 2016, I could not walk so I used the walker, and I went to the hospital and my blood sugar level was 350.”* P5AB2

Some received a diagnosis during pregnancy. Almost all respondents reported a family history of diabetes. Respondents reported difficulties with adhering to the medication treatment regime post-diagnosis, not taking medication (metformin or insulin) when blood sugars were perceived to be good, and taking the medication when they felt symptoms such as fatigue. Related to that, respondents reported that they were afraid of taking too much medication and believed that it would affect their kidneys, or that they could become addicted to insulin injections.

*“If I take the medicine regularly, my kidney will hurt, I am afraid of that. But if I feel fatigue, then I take it. Sometimes I boil water with pandan leaves and drink the water, I feel strong. But I have not done it so often.”* (P7BNA)

Some reported replacing metformin pills with alternative natural products based on the belief that these would also lower blood sugar levels.

“*People said, pagar-pagar leaf is good, so if I drink these boiled leaves, then I don't take the pill anymore.*” (P1AB1)

Respondents also reported efforts to change their diet by replacing rice or sugar with products perceived to have less effect on blood sugar levels. Some also tried to eat less but had problems maintaining their diet.

*“I substitute the sugar to Tropicana slim (low glucose sugar), I reduce my meal like I was told in Prolanis. We often feel hungry, so I prepare bread at 10 am. Sometimes we crave for something sweet, once we have it, it is hard to stop.”* (P7BNA)

Some of the information provided by Prolanis is difficult for patients to comprehend. Many respondents found presentations and videos more engaging than brochures. In addition, many respondents had difficulties reading the information in the brochures.

“*Honestly, brochures we don't like, because we are lazy to read, it is better to have a PowerPoint presentation.*” (P5AB2)

“*Sometimes playing the video is also good, so it will not be so boring.*” (P3BA)

## Discussion

Diabetes and other NCDs have become a pressing issue for health systems in LMICs. From our data on individuals with type 2 diabetes in Banda Aceh and Aceh Besar, it is evident that current measures to treat type 2 diabetes are not meeting their needs. We found that the average person with diabetes had HbA1c levels of almost twice the level that would be taken to indicate pre-diabetes. There is clear evidence that elevated HbA1c levels are highly predictive of diabetes-related morbidity and mortality [9–11], which is also reflected in the large number of people who reported an existing diabetes complication in our data. Similarly, we found high rates of obesity, hypertension and dyslipidemia, making it more likely that those without existing diabetes complications will develop complications in the near future. Given the high HbA1c levels indicative of largely unmanaged diabetes, even relatively minor changes to the treatment regime could have important effects. For example, a recent study in the same area evaluated the usability of novel smartphone-based test devices in Puskesmas (Rhode et al.: Smartphone-based point-of-care diagnostics in primary health care to monitor HbA1c levels in patients with diabetes: a validation study, unpublished manuscript). These could be a cheap and effective alternative to laboratory-based tests for HbA1c levels and lipid profiles which are currently not available at all Puskesmas, or are only provided irregularly, preventing their use for continuous monitoring to inform treatment decisions. The qualitative findings indicate that people with diabetes often only engage with the health system after experiencing diabetes symptoms, which is too late. There is evidence from another study carried out in the same region, that relatively simple interventions, such as SMS reminders to attend health screenings, can increase healthcare-seeking behavior [12]. Similar interventions have also been shown to improve diabetes care and might be used to increase healthcare seeking in people who have diabetes, or are at risk of developing diabetes, in our setting [13].

We further found that existing governmental programs suffer from low participation rates, low engagement of participants beyond the use of medical services offered during these meetings, and materials that are difficult for many participants to comprehend or read. One way to increase participation could be to align meeting times with the schedules of working-age adults. Another improvement could be to design meetings and information material to be interesting, accessible and actionable for participants.

We find that patients find it difficult to adhere to recommended treatment and lifestyle advice and use alternative treatments to replace or complement their medical therapy. False beliefs about diabetes are also common in other settings [14, 15]. To address false beliefs, an intervention must respect local circumstances and beliefs, but has to overcome myths that interfere with treatment of patients who are in dire need of insulin but do not take it. Our mixed-methods approach reveals that structural problems are impeding the effectiveness of diabetes-management programs in Aceh, Indonesia. Relatively simple interventions based on scientific evidence, derived from other studies in the region, could be used to improve care-seeking behavior and the health information that is available to people with diabetes.

## Data Availability

The data will be made available for non-profit scientific research purposes upon reasonable request to the corresponding author. Consent for data sharing with other researchers has been obtained from the participants. Interested researchers will need to provide us with their name, affiliation, and the goal of the research project for which they want to use the data. The data will then be transferred electronically in encrypted password-protected files with the password sent separately.
